# Randomised controlled trial of the effects of physical activity *feedback *on *awareness *and *behaviour *in UK adults: the FAB study protocol [ISRCTN92551397]

**DOI:** 10.1186/1471-2458-10-144

**Published:** 2010-03-18

**Authors:** Clare Watkinson, Esther MF van Sluijs, Stephen Sutton, Theresa Marteau, Simon J Griffin

**Affiliations:** 1 MRC Epidemiology Unit, Institute of Metabolic Science, Addenbrookes Hospital, Box 285, Hills Road, Cambridge, CB20QQ, UK; 2 General Practice & Primary Care Research Unit, Institute of Public Health, Forvie Site, Robinson Way, Cambridge, CB2 0SR, UK; 3 King's College London, Psychology & Genetics Research Group, Institute of Psychiatry, 5th Floor Thomas Guy House, Guy's Campus, London, SE1 9RT, UK

## Abstract

**Background:**

While there are increasing data implicating poor recognition of physical inactivity as a potential barrier to healthy behaviour change, the efficacy of feedback to promote physical activity is uncertain. Using a randomised controlled trial nested within a population-based cohort study, we plan to test three variations of physical activity feedback against a control group. Our primary objective is to assess the efficacy of physical activity feedback in promoting physical activity behaviour change. Secondary objectives are to determine the influence of feedback on physical activity awareness and cognitions, and to compare behavioural effects by type of feedback.

**Methods/Design:**

We aim to recruit 500 healthy participants aged 30 to 55 years from the ongoing Fenland Study (Cambridge, UK). Following careful phenotyping during baseline measurement (anthropometric, clinical, body composition and fitness measurements, as well as questionnaires assessing self-reported and self-rated physical activity, psychosocial correlates of physical activity behaviour, diet, lifestyle and general health), participants wear a combined heart rate and movement sensor (Actiheart^®^) for six continuous days and nights. After receipt of the physical activity data (around 2 weeks later), participants are randomly allocated to either a control group (no feedback) or one of three types of personalised physical activity feedback ('simple', 'visualised' or 'contextualised'), and complete repeat measures of self-rated physical activity and psychosocial correlates. Approximately five weeks after receiving feedback, all participants wear the Actiheart^® ^for another six-day follow-up period and complete repeat questionnaires. Values at outcome, adjusted for baseline, will be compared between randomised groups.

**Discussion:**

Given the randomised trial design and use of objective measure of physical activity, this study is likely to provide valuable insights into the efficacy of a feedback intervention in changing physical activity behaviour, as well as the psychological mechanisms involved.

**Trial Registration:**

Current Controlled Trials: ISRCTN92551397

## Background

Low levels of physical activity have been associated with a variety of health problems, including mortality, cardiovascular disease, metabolic disorders and certain forms of cancer [1]. Two-thirds of UK adults do not meet government targets for physical activity [2] and effective strategies to promote active lifestyles are still lacking [3,4]. Even where interventions have had positive results, recent reviews show that effect sizes are generally small and short-lived [3]. It is unclear whether the absence of anticipated outcomes in intervention studies is due to failure to target key determinants and mediators, inadequate execution of an intervention or inexact measurement of the outcome [3].

One possibility is that sedentary individuals do not perceive themselves as such, incorrectly believing themselves to be active. Unlike dichotomous behaviours such as smoking, physical activity is complex, spanning multiple planned, incidental and habitual activities over a 24-hour period. Consequently, thresholds of sufficient and insufficient activity may be unclear [5]. Evidence to date suggests that up to 60% of adults who do not currently meet the recommended guidelines for physical activity overestimate their own level [6]. Moreover, only 27% report a positive intention to change behaviour, compared to 43% among those who accurately assess their inactivity [6]. Despite being at greatest risk of health problems, those who fail to recognise their inactivity are unlikely to perceive a need to change and may be less susceptible to health promotion strategies.

Studies on the correlates of misperceptions about health behaviours suggest correlations with anthropometric characteristics and styles of interpersonal comparisons. People who erroneously classify themselves as adequately active are more likely to compare themselves with those perceived to engage in the same or lower levels (downward comparison) for example, and to rate their own behaviour as healthier (optimistic bias) [7,8]. Studies also show that overestimation is associated with favourable indicators of health. Those with a lower body mass index (BMI) or body fat %, or with a more positive general perception of their health, more often assume that their physical activity is sufficient or high [5,6,9]. Such findings could help identify and target individuals at risk of such misperceptions.

Physical activity awareness (defined as the agreement between self-rated and actual activity level according to current guidelines) has rarely been studied as a determinant of healthy behaviour change. The Precaution Adoption Process Model identifies awareness of personal risk behaviour as an important step toward behaviour change, and posits that people are only expected to consider changing their behaviour when they become aware that *they personally *engage in too little physical activity and are potentially putting their health at risk [10]. Measurement and feedback may help to achieve this and have been shown to increase both awareness of health behaviour and intentions to change that behaviour [11,12]. Along similar lines, self-regulation theories consider self monitoring (a particular type of measurement and feedback) to be an essential element of behavioural self-regulation [13,14]. Indeed, a recent review of studies including pedometer interventions demonstrated consistent associations between the use of pedometers and increased physical activity [15].

Little is known about the effects of external feedback on physical activity awareness, intentions and behaviour, and even less about the efficacy of different types of feedback [16]. Of the evidence that is available, the majority comes from risk communication research and hypothetical vignette studies where the effects of feedback are primarily evaluated on the ability to influence perceptions of risk or intentions to change behaviour. For example, studies in tanning booth users [17] and smokers [18] show that people receiving personalised visual images of their disease or risk (a photograph that highlighted UV damage on the face and an ultrasound image of atherosclerotic plaque build-up in their carotid artery, respectively) are more likely to change their behaviour than those provided with written or verbal feedback. In addition, research also highlights that individually tailored interventions are more likely to be read, saved, remembered and discussed [11,19,20] and that goal setting in combination with self-monitoring is more successful [15,21,22]. However, to our knowledge no study has objectively measured change in health behaviours. A recent empirical review identified only eight randomised trials that investigated the effects of 'biomarker' feedback (biological indices of physical harm, disease, or increased disease risk) on motivation and intention to change health-related behaviour, or behaviour change itself [23]. Of those identified, only one examined physical activity behaviour. While there was some indication that feedback may increase motivation to change behaviour, this was limited by a reliance on imprecise measures of behaviour [24].

Importantly, the potential negative effects of feedback have also not been adequately addressed [25]. Many people who undergo a physical assessment receive results that lie within the normal or recommended range. Little is known about the impact of these 'desirable' results on future health beliefs and behaviour. While some people may be motivated to maintain their current status, others may be falsely reassured, perceiving less need to engage in health-promoting behaviours [26,27]. Conversely, undesirable feedback may trigger denial, threat minimisation or fatalistic attitudes, impeding an active role in health behaviour change [28]. Of seven studies reporting on the impact of cholesterol screening in a recent systematic review, six reported negative consequences for acceptance of risk caused by receipt of a high-risk result [25].

The present study will be the first to combine a randomised controlled trial design, objective outcome assessment and population-based sample to explore the effects of feedback on physical activity awareness, intentions and behaviour. We draw on relevant theories to select psychological measures with evidence of predictive ability to enable us to identify possible moderators and mediators of behaviour change. We will test three feedback types: simple, visual or contextualised. Our main aim is to assess the influence of personalized and normative physical activity feedback on free-living physical activity physical activity awareness and cognitions by comparing outcomes in three intervention groups (collectively and individually) against a control group. Our secondary research questions are a) which cognitions mediate the intervention effect, and b) whether potential effect(s) differ by feedback type.

## Methods/Design

### Design

The Feedback, Awareness and Behaviour study (FAB) is a randomised controlled trial with randomisation of 500 participants of the Fenland Study to either no feedback (control group) or to 'Simple', 'Visual', or 'Contextualised' physical activity feedback (intervention groups).

### Recruitment

#### The Fenland Study

The Fenland Study is an ongoing population-based cohort study investigating the influence of diet, lifestyle and genetic factors on the development of diabetes, obesity and other metabolic disorders http://www.mrc-epid.cam.ac.uk/Research/Studies/Fenland/index.html. Residents of Cambridgeshire (East of England, UK) aged 30-55 years registered at participating general practices (GP) are eligible to take part. Potential participants are excluded from the Fenland study by their GP if they have been diagnosed with diabetes, have a terminal illness with a prognosis of less than one year, suffer from a psychotic illness, are pregnant or lactating, or are unable to walk unaided. Recruitment operates via a pre-defined sampling frame (a list of patients meeting the inclusion criteria provided by all participating GPs prior to commencement of the study), whereby potential participants are assigned a study ID number and contacted in a random order. GPs approach potential participants via letters enclosing an information sheet, reply slip and freepost reply envelope. Individuals who return positive replies are contacted by the study office to arrange an appointment for them to attend a measurement facility. Written confirmation of the appointment is sent two weeks before, the Fenland informed consent form is signed on the testing day. Currently, around 30% of adults registered with participating general practices in the Cambridgeshire Primary Care Trust have agreed to take part.

#### The FAB Study

Between September 2007 and August 2008 all Fenland participants were invited to take part in the FAB Study via a letter and information sheet included in their appointment confirmation packs. These explain that we are looking for a small number of participants who would be willing, in addition to their standard visit, to complete some further measures. Full details are provided in the Information Sheet, along with a short summary of the aim ('to investigate the effects of the Fenland study experience on participants and to help us understand the best way of providing people with feedback on their health'). Those who agree to participate are asked to sign an additional consent form at the beginning of their testing day.

### Study flow/procedures

#### Baseline

**Figure 1:**
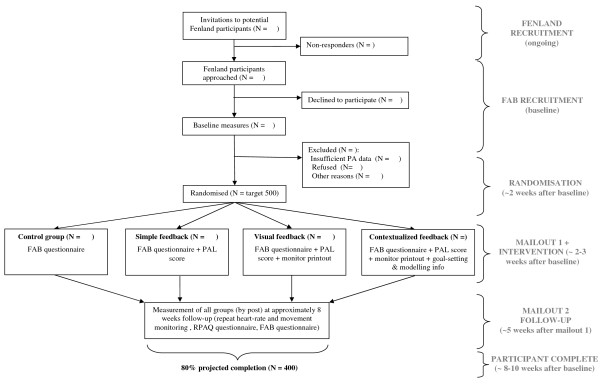
**Flow of participants in FAB study**.

Trial design and participant flows are shown in Figure 1. Immediately after giving informed consent (and prior to any Fenland testing) participants are asked to complete the FAB baseline questionnaire measures. On the testing day, Fenland participants undergo a range of anthropometric (e.g. height, weight, hip and waist circumference), clinical (e.g. blood pressure), body composition (e.g. body fat percentage and distribution using ultrasound and dual energy x-ray absorptiometry) and fitness measurements (heart rate (HR), movement and oxygen consumption at rest and during a sub-maximal treadmill test). They also complete questionnaires on diet, physical activity, medical history and general lifestyle. In addition, an oral glucose tolerance test is administered and two blood samples are taken to assess glucose levels and blood lipids. At the end of the appointment, which takes an average of 3 to 3.5 hours, participants are fitted with a combined movement sensor and HR monitor (Actiheart^®^, CamNtech, Cambridge, UK [29]). This is worn for six days and nights and returned to the measurement facility in a prepaid special delivery envelope.

#### Randomisation

Once the Actiheart^® ^monitor has been successfully downloaded and all relevant baseline data are available, participants are randomly allocated to either the control group or one of three feedback groups. Randomisation is carried out using a statistical minimisation programme (overseen by a statistician) on the basis of age (<45, ≥ 45 years), gender (male/female), baseline physical activity level (PAL), an expression of the ratio of total energy requirements to basal metabolic rate over a 24-hour period (<1.63, ≥ 1.63), BMI (<27, ≥ 27 kg/m^2^) and glycosylated haemoglobin (Hba1c: <5.4, ≥ 5.4%), and is carried out independently of those undertaking baseline and follow-up measurements at the testing sites. Minimisation cut-offs were derived from an analysis of mean and median values in the first 1000 Fenland participants measured. Participants for whom sufficient baseline physical activity data is not available (less than 3 full days or 35 hours in total) are excluded from the study. Current data from the Fenland study suggest that this applies to fewer than 10% of participants. Since the FAB study is a trial of the impact of feedback of information, it is not possible to conceal group allocation from participants.

#### Mailout 1 (approximately 2 weeks after the testing day)

After receipt of the physical activity data, all participants are sent the second FAB questionnaire and participants allocated to one of the feedback groups receive their personal physical activity feedback. They are asked to read through the feedback and check that they have understood it before completing the questionnaire. All groups are asked to return their completed questionnaires by freepost envelope. A reminder letter, along with a second copy of the questionnaire, is sent if responses are not received within 2 weeks. The timing of Mailout 1 is dependent on the speed with which the first Actiheart^® ^monitor is returned, but will usually occur around 2 weeks after baseline measurement.

#### Mailout 2 (approximately five weeks after Mailout 1)

Approximately five weeks after Mailout 1 is posted, participants are contacted by telephone to arrange sending out their second measurement pack (Mailout 2). This includes an Actiheart^® ^monitor, two questionnaires (FAB questionnaire and self-reported physical activity) and full instructions about how to attach the monitor correctly. They are asked to wear the monitor for another period of six days and nights and to return it with their completed questionnaires using the prepaid special delivery envelope provided. The purpose of the telephone call is to ensure that people will be able to wear the monitor in the near future. If this is not possible, a more convenient timing will be arranged.

We decided on a five-week (post-intervention) follow-up period to allow sufficient time for the dissipation of early novelty responses and thus detect behaviour change of a more sustained nature. Allowing one week for information to 'sink in', it also matches the reference period covered by physical activity questionnaire (one month). Mailout 1 is chosen as the baseline index point for calculating Mailout 2 posting dates in order to control the period of time between intervention and outcome measurement as much as possible. In total, we aim that participation in the FAB study lasts for approximately 8-10 weeks from a participant's initial Fenland testing day to completion of follow-up, but this may vary between participants due to monitor re-wear and delays in sending out Mailout 2. Overall duration and stage-specific duration are recorded during the study in order to ensure equal average follow-up time between the groups.

### Intervention

**Figure 2:**
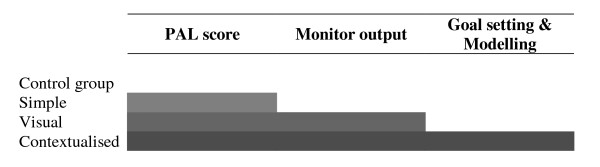
**Distribution of feedback components across the four trial groups in the FAB study**.

The content of each feedback type has been chosen to reflect promising approaches identified in the recent literature. To facilitate the isolation of effect estimates for individual feedback components, each feedback level is built on the previous one to create an ordered categorical variable based on simple feedback as its most basic level (see Figure 2).

#### Simple Feedback

Participants randomised to this group receive a short definition of physical activity; a summary of its health benefits; and a brief reminder of current guidelines (see additional file 1, Appendix A). In addition, they are informed of their average PAL across the period during which they wore the Actiheart^® ^monitor at baseline. This is calculated using Actiheart^® ^software, and is provided alongside a simple table showing the FAO/WHO/UNU reference categories (Table 1) [30].

**Table 1:** Reference values for physical activity levels (PAL) as published by FAO/WHO/UNU [30]

PAL Value	Description
Less than 1.2	Bed rested: Most likely when in care of others
1.2 to 1.55	Low activity level: Sedentary lifestyle.
1.55 to 1.71	Medium activity level: Occasionally active. Typical office work.
1.71 to 1.95	High activity level: Some manual work and/or regular exercise
Greater than 1.95	Very high activity level: A fair amount of manual work or exercise training.

#### Visual Feedback

As mentioned previously, studies have indicated that people who are shown personalised visual images of their disease or risk are more likely to change their behaviour than those provided with written or verbal feedback. In one study, students shown a photograph that highlighted UV damage on their face reported less tanning booth use at follow-up than students not shown such a photograph, though both received verbal and written risk information [17]. Another study found that showing smokers an ultrasound image of atherosclerotic plaque build-up in their carotid artery, together with an image of a disease-free artery, increased perceptions of risk and intentions to stop smoking compared to those who received routine verbal feedback [18].

In the context of the Fenland study, the nearest approximation to 'visual imagery' is the output generated from the Actiheart^® ^software (see additional file 1, Appendix B). Participants randomised to 'Visual Feedback' receive a modified version of this output (consisting of a series of graphs on one side of A4), alongside their 'simple feedback'. Each graph represents a single day of measurement along a 24-hour x-axis and plots a graphical record of the participant's HR and movement counts for each day they wore the sensor, briefly explained in their feedback sheet. We anticipate that this will allow participants to see how their HR and movement vary -or do not vary- at different times of the day or week, and to correlate specific activities they remember undertaking with corresponding peaks or troughs in the lines. Participants also receive example printouts of each PAL value described in the reference table, each one illustrating a HR and movement pattern typical of this PAL value.

#### Contextualised Feedback

Studies have indicated that goal setting is associated with more successful weight management [21,22] and significant increases in daily pedometer counts [15], and that people who set personal goals tend to use positive behavioural strategies over negative ones [31,32]. On this basis, the third level of feedback ('Contextualised Feedback') aims to provide tailored goal setting and modelling information (see additional file 1, Appendix C). In addition to the 'simple' and 'visual' components, it includes estimates of the added PAL value of familiar activities (e.g. housework, walking or cycling) calculated for different durations (1 hour, 2 hours etc). It also incorporates a short fictional gender-specific vignette based on a 'typical' Fenland participant (aged 35-50), designed to address physical activity misperceptions and encourage behaviour change. Although the actual content was identical, 'Jenny' was used for the female version and 'John' for the male version to promote identification with the character.

The duration of each activity necessary to increase average daily PAL by 0.1 or 0.2 was calculated using the updated Compendium of Physical Activities [33]. Average daily resting energy expenditure (REE) was taken as equal to 1392 METmins/day (REE = 1 × 960 METmins/day + 0.9 × 480 = 1392 METmins/day).

#### Pilot testing

Materials were pilot tested with twenty Fenland participants, who were asked to read through the example feedback a couple of times before taking part in a short structured interview. This aimed to explore their understanding, attitude, opinions and preferences in relation to the material presented, and minor revisions were made on the basis of the results. To address the confusion expressed by some participants about the main determinant of PAL (movement or HR), we included a brief explanation in all feedback types. We also clarified the connection between the participant's PAL result, the reference table and the example Actiheart^® ^printouts in the visual feedback. Lastly, we removed unnecessary details from the Actiheart^® ^graphs (e.g. HR and movement scales on the y-axis) and enlarged the image to facilitate comprehension.

### Measurements

#### Objectively-measured physical activity

All trial measures and their timing are shown in Table 2. The main outcome measures are 1) physical activity energy expenditure per kg of fat free mass/minute (PAEE) and 2) total daily movement counts (DPA), measured via individually-calibrated HR and movement monitors (Actiheart^®^) [29]. Participants are asked to wear the monitor for six days and nights continuously, and to carry on with all normal activities during this time. The Actiheart^® ^is a non-invasive, single-piece combined monitor, which weighs less than 8 g, is 7 mm thick (33 mm in diameter), waterproof and worn on the chest attached to standard ECG electrodes. It is capable of measuring acceleration, HR, HR variability, and ECG amplitude for a set time resolution. The monitor is convenient and discreet to wear, helping to reduce the potential Hawthorne effect (behavioural modification caused by the act of being observed) [34,35]. Participants are asked to complete an diary sheet, noting down the date and time they a) started wearing the monitor, b) removed it (along with the reason), and replaced it again, and c) completed measurement.

**Table 2:** FAB trial measurements and their timing

	Baseline visit	Mailout 1 (~2 wks post- baseline)	Mailout 2 (~5 wks post- Mailout 1)
**Main outcome**			
1. PA - 6-day HR and movement monitoring (Actiheart^®^)	✓		✓
**Other outcomes**			
2. PA awareness	✓		✓
3. Fenland measures (anthropometry, body composition, fitness)	✓		
4. Self-reported PA	✓		✓
5. Self-rated PA:^a^			
i. Absolute (WHO categories)	✓	✓	✓
ii. Relative (peer comparison)	✓	✓	✓
iii. According to guidelines (CMO)	✓	✓	✓
6. Confidence in self-rated PA^a^	✓	✓	✓
7. PA Subjective norms^a^	✓	✓	✓
8. Worry/concern about PA^a^	✓	✓	✓
9. Perceived behavioural control/self efficacy^a^	✓	✓	✓
10. Behavioural beliefs^a^	✓	✓	✓
11. Perceived importance of physical activity for health^a^	✓	✓	✓
12. Intention to change PA^a^	✓	✓	
13. Time orientation (concern about current/futureconsequences)^a^	✓		

PA: Physical activity; WHO: World Health Organisation; CMO: Chief Medical Officer

^a^: included in FAB questionnaire

HR response to a sub-maximal exercise test is established during the testing day and is used for individual calibration of the Actiheart^® ^[36], and branched equation modelling is utilised to estimate PAEE [37]. This approach has high validity for estimating the intensity of physical activity [38] and overcomes some of the key limitations associated with either accelerometers or HR monitors alone [29].

#### Questionnaire measures

Self-reported physical activity is measured using the Recent Physical Activity Questionnaire (RPAQ). This quantifies physical activity in four domains (work, travel, recreation and domestic life) over the preceding month. A validation study using doubly labelled water as the golden standard has shown the RPAQ to be valid in ranking individuals according to their energy expenditure [39].

Self-rated physical activity is measured at all time-points using three different reference standards: 'Absolute' (PAL categories defined by FAO/WHO/UNU [30]); 'Relative' (peer comparison); and 'Recommended' (according to CMO guidelines [40]) (see Table 3). A question to assess the participant's confidence in their answers to these three questions is also included: "Overall, how confident do you feel about your answers to questions 1 to 3?" (*very/moderately/somewhat/not at all*).

**Table 3:** Description of the three types of self-rated physical activity measured in the FAB study.

Category	Reference standard	Question used (answer categories)
Absolute	PAL categories defined by WHO categories [30]	On average, which category do you believe best describes your general level of physical activity? (Bed-rested/Low/Medium/High/Very High).

Relative	Peer comparison	In your opinion, compared to other people of your age and sex, how physically active are you? (Much less/A little less/About the same/A little more/Much more).

Recommended	CMO guidelines [40]	According to national recommendations people should be active at a moderate intensity (e.g. brisk walking) for at least 30 minutes per day at least 5 days a week. Please indicate whether you think you achieved this level of activity over the last month. (Yes/No).

PAL: Physical activity level; WHO: World Health Organisation; CMO: Chief Medical Officer

**Figure 3:**
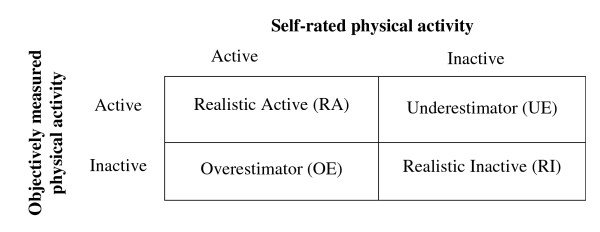
**Method of classification of participants into awareness categories**.

Physical activity awareness is defined as the agreement between 'recommended' self-rated activity and objectively-measured physical activity according to current guidelines [40]. The 'recommended' reference standard was considered most relevant to public health research as it is the primary reference point for most physical activity interventions and health promotion messages [40]. Objective physical activity is defined on the basis of average PAL. Participants are classified as either active (PAL ≥ 1.7) or inactive (PAL<1.7) [30,41]. Self-rated and objectively-measured physical activity levels are then grouped in a 2 × 2 table to create four awareness categories: 'Realistic Actives', 'Realistic Inactives', 'Overestimators' and 'Underestimators' [6] (Figure 3).

Cognitive predictors of physical activity hypothesized to be directly associated with behaviour change are measured at all three time points [42]. Questions were drawn from the previously validated ProActive study questionnaires, which were based on the Theory of Planned Behaviour [43], and amended where appropriate [44,45]. Items are measured on a Likert scale ranging from 1 (*strongly disagree*) to 5 (*strongly agree*), and cover perceived adequacy ('I do enough physical activity to stay healthy'), subjective norms ('Most people who are important to me would want me to be more physically active'), perceived behavioural control/self efficacy ('I am confident that I could be more physically active in the next two months, if I wanted to'), behavioural beliefs ('If I was more physically active in the next two months, it is likely that my fitness would improve/my appearance would improve/I would feel better/my health would improve'), perceived importance for health ('Physical activity is important for maintaining good health'), and intention to change ('I intend to be more physically active in the next two months'). Worry and concern about physical activity is measured via a separate 5-point Likert response scale (*not at all/rarely/sometimes/often/almost all the time*) using two items ('During the past two weeks, how often have you thought about your level of physical activity/how often have thoughts about your level of physical activity affected your mood?'). Given previously suggested associations with other health-related behaviours [46,47], a validated 9-item time-orientation measure (concern about current/future consequences) [48] is also completed at baseline via a 5-point scale ranging from 1 (*very unlike me*) to 5 (*very like me*).

Questionnaires were piloted over two weeks at the Fenland testing facility in Ely, where participants' experiences and reactions were recorded via a brief structured interview with a member of the FAB study team. A few minor changes to question wording were made on the basis of respondent feedback, but overall comprehension was high and questions were rated as clear and user-friendly.

### Data analyses

Analyses will be undertaken on an intention-to-treat basis. The main experimental comparison is receipt of physical activity feedback (intervention) versus no feedback (control), with PAEE and DPA as the principal outcomes and self-reported physical activity, awareness and cognitions as secondary outcomes. Values at outcome, adjusted for baseline, will be compared between randomised groups. Gender, baseline physical activity and baseline awareness will be investigated as potential moderators of the intervention effect. In addition, we will conduct sensitivity analyses assuming a range of potential outcomes for non-completers informed by available baseline and interim data on this group. Non-completers will have multiple data imputed with a 'missing at random' assumption and with sensitivity analyses to represent optimistic and pessimistic scenarios for drop out. To assess mediating effects, a product-of-coefficient test will be used [49]. A secondary dose-response analysis will compare each feedback type ('Simple', 'Visual' or 'Contextualised') with the control condition and with each other.

### Sample size

Calculations were undertaken for a comparison between two equal-sized groups. Although the primary FAB analysis involves combining intervention groups and comparing them collectively against the control condition (intervention-control ratio = 3:1), secondary analyses compare each intervention group individually with the control group (intervention-control ratio = 1:1) and each other, and will therefore require additional power. Calculations are therefore based on the secondary analyses.

Estimates are taken from the ProActive trial [50], which used a comparable population, age-group and primary outcome to those proposed here. Participants mean (standard deviation, SD) PAEE at baseline in this study was 0.116 (0.076) kJ/kgFFM/min. For a comparison between two groups, 100 participants per group completing follow-up would allow detection of a difference of 0.03 kJ/kgFFM/min in physical activity energy expenditure (which equates to approximately 225 to 300 Kcals, or roughly 20 mins brisk walking per day) with 80% power at the 5% significance level. However, by adjusting for baseline values we obtain greater precision. The correlation between baseline and follow-up PAEE in ProActive was 0.58, meaning that 100 participants per group would allow detection of a difference of 0.025 kJ/kgFFM/min (0.33 SD). Thus we aim to randomise a total of 500 participants, with the expectation that 400 (80%) would complete follow-up.

### Data management

Each participant is assigned a unique numeric identifier code at the beginning of the Fenland study so that they can be tracked without reference to personal information and this will be continued to be used for the FAB study. As per usual Fenland procedures, all personal data is stored on an encrypted drive, and links to personal information are available only to the Fenland and FAB study coordination teams. Consent forms and questionnaire data are double-entered and stored in locked filing cabinets in secure Entacard-protected sites.

### Ethics

Full ethical approval for the FAB study was obtained from the Cambridge Local Research Ethics Committee on 4^th ^June 2007 (reference number 07/Q0108/79). The study was registered under trial number ISRCTN92551397.

## Discussion

There is increasing evidence that poor recognition of physical inactivity may be an important barrier to healthy behaviour change. Compelling observational data suggest that in terms of physical activity attitudes and intentions, people who incorrectly believe that their physical activity is adequate are comparable to those who achieve the recommended guidelines [5,9,51] and thus resemble a group that public health interventions may not be able to reach. The FAB trial is designed to explore the effect of feedback on physical activity awareness, cognitions and behaviour in a population-based sample. In addition to estimating efficacy, it will provide information on possible psychological mechanisms of behaviour change. FAB has the potential to establish the extent to which increasing the accuracy of peoples' self-perceptions of physical activity might facilitate healthy behaviour change. However, it will also allow for the assessment of the risk for false-reassurance.

Our trial has been designed to address the limitations of previous work in the area of physical activity awareness and feedback. The use of a combined HR and movement sensor to asses physical activity overcomes the limitations associated with self-reported physical activity behaviour or intentions and is more accurate than either HR monitoring or accelerometry alone [29]. By drawing on relevant theory and evidence, we have chosen covariates with evidence of predictive ability for measurement at baseline and follow-up. These are expected to facilitate identification of possible moderators and mediators of behaviour change.

FAB is the first randomised trial of the effects of measurement and feedback on physical activity awareness, cognitions and behaviour using an objective measure of behaviour change and a population-based sample. Findings will be relevant to future studies that try to explain and change health behaviours in general and those on misperceptions about health behaviours in particular. Overall, it is expected that this will help inform the design of future preventive programmes promoting physical activity.

## Competing interests

The authors declare that they have no competing interests.

## Authors' contributions

SG, EvS and CW defined the research question. All authors participated in the design of the trial, feedback intervention and measures. CW drafted the manuscript; all authors were involved in critical revisions and have read and approved the final manuscript.

## Pre-publication history

The pre-publication history for this paper can be accessed here:

http://www.biomedcentral.com/1471-2458/10/144/prepub

## Supplementary Material

Additional file 1 **Examples of the FAB feedback**. Examples of the three types of physical activity feedback provided in the FAB study: Simple (Appendix A), Visual (Appendix B), and Contextualised (Appendix C). Click here for file
