# Supplementary material for: Randomised controlled trial of the effects of physical activity feedback on awareness and behaviour in UK adults: the FAB study protocol [ISRCTN92551397]
Source: BMC Public Health. 2010 Mar 18;10:144. doi: 10.1186/1471-2458-10-144 (PMC2859395; doi:10.1186/1471-2458-10-144)
Supplement: Additional file 1 — Examples of the FAB feedback. Examples of the three types of physical activity feedback provided in the FAB study: Simple (Appendix A), Visual (Appendix B), and Contextualised (Appendix C). [file 1471-2458-10-144-S1.DOC]

Appendix A: “Simple” feedback


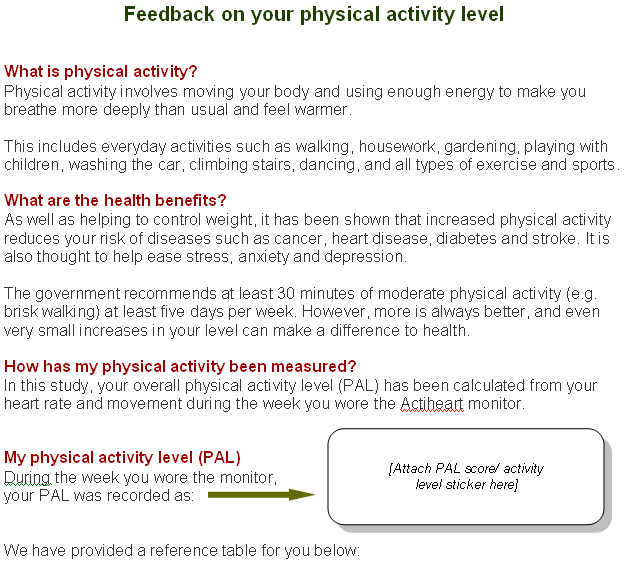


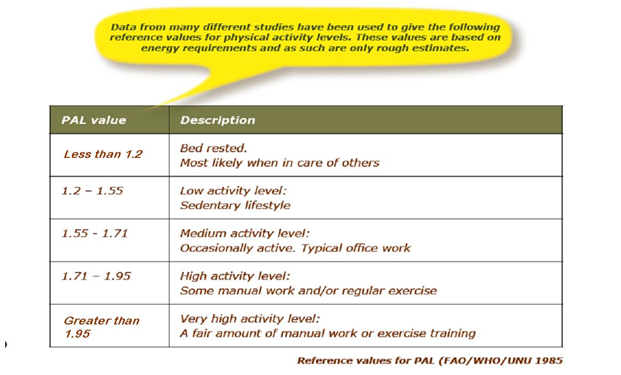


**Appendix B: “Visual” feedback**


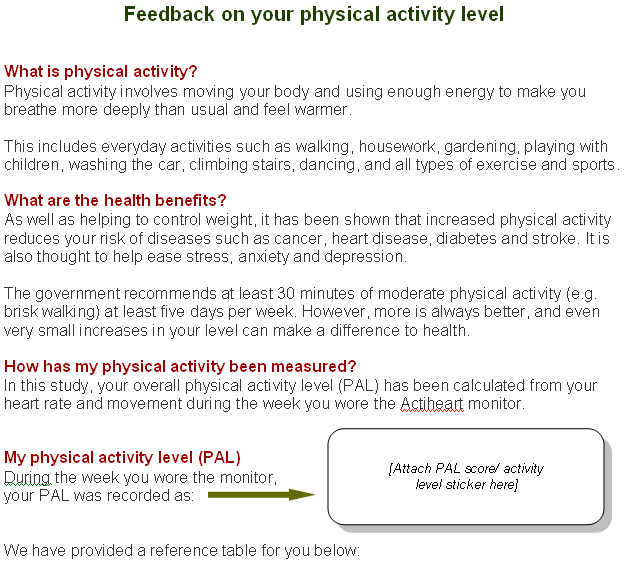


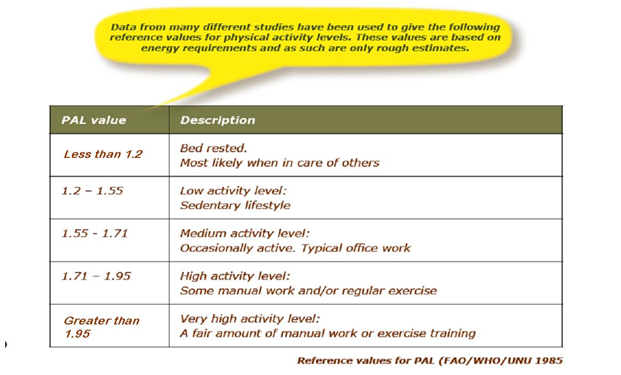


**
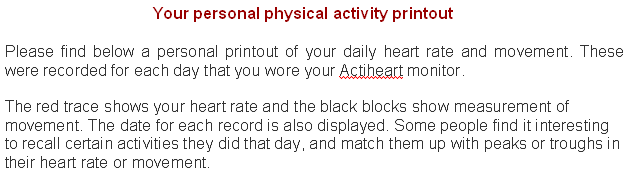
**

**
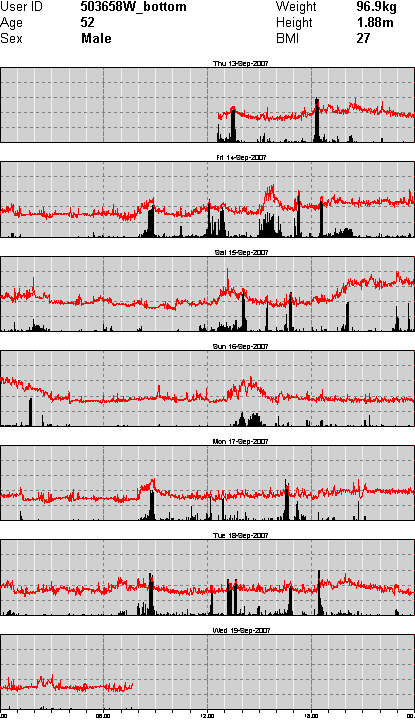
**

**Examples from other participants**

The examples below show printouts of each level of physical activity described in the reference table (page 1).

The examples are taken from a selection of participants. Each separate graph represents a single day of measurement, and is taken from a different person to show a heart rate and movement pattern typical of that activity level.

You might find it useful to compare your personal daily graphs to these examples. Higher levels of physical activity are indicated by a high or varied heart rate or more black areas.

**
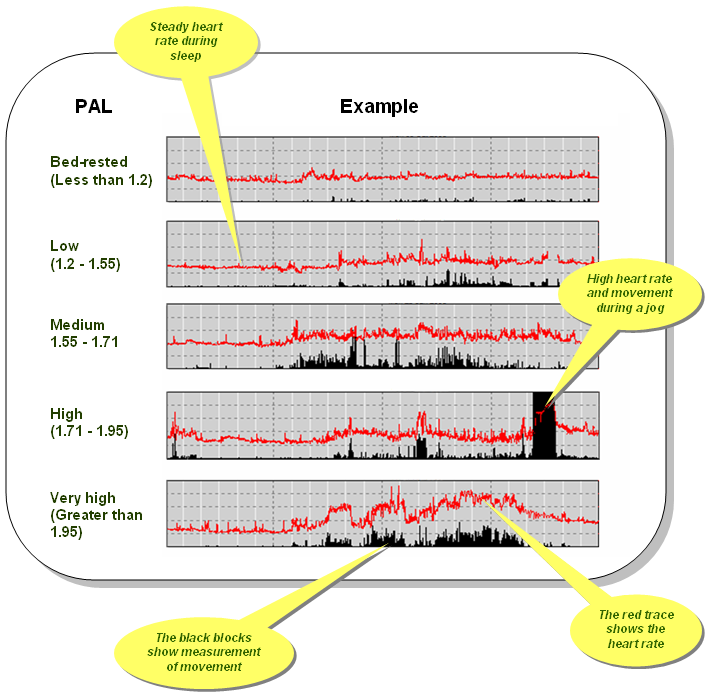
**

**Appendix C: “Contextualised” feedback**


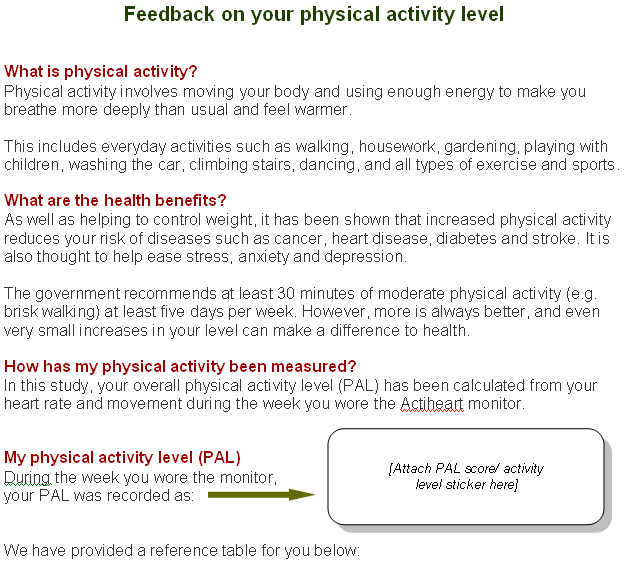


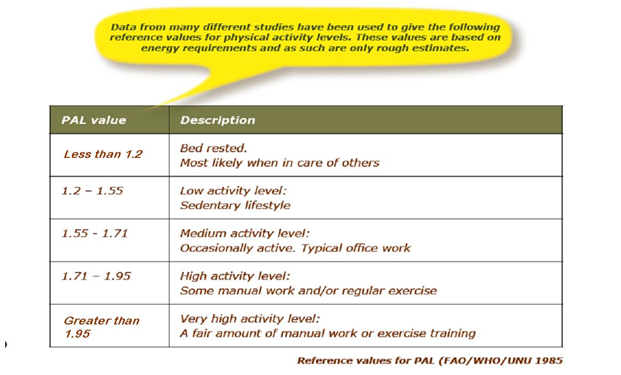


**
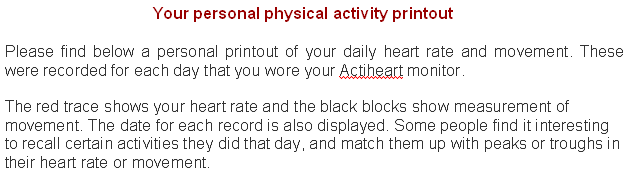
**

**
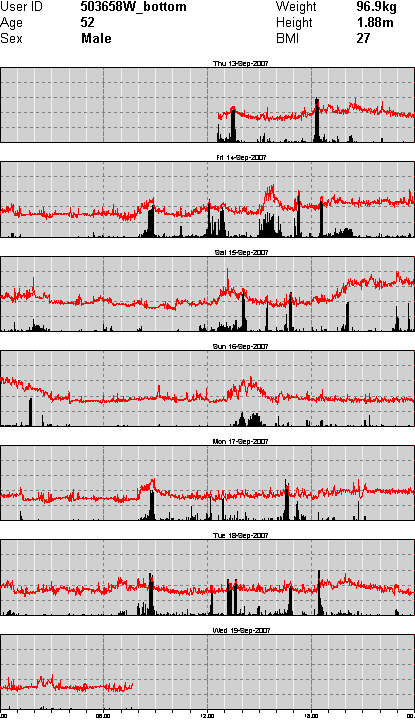
**

**Examples from other participants**

The examples below show printouts of each level of physical activity described in the reference table (page 1).

The examples are taken from a selection of participants. Each separate graph represents a single day of measurement, and is taken from a different person to show a heart rate and movement pattern typical of that activity level.

You might find it useful to compare your personal daily graphs to these examples. Higher levels of physical activity are indicated by a high or varied heart rate or more black areas.

**
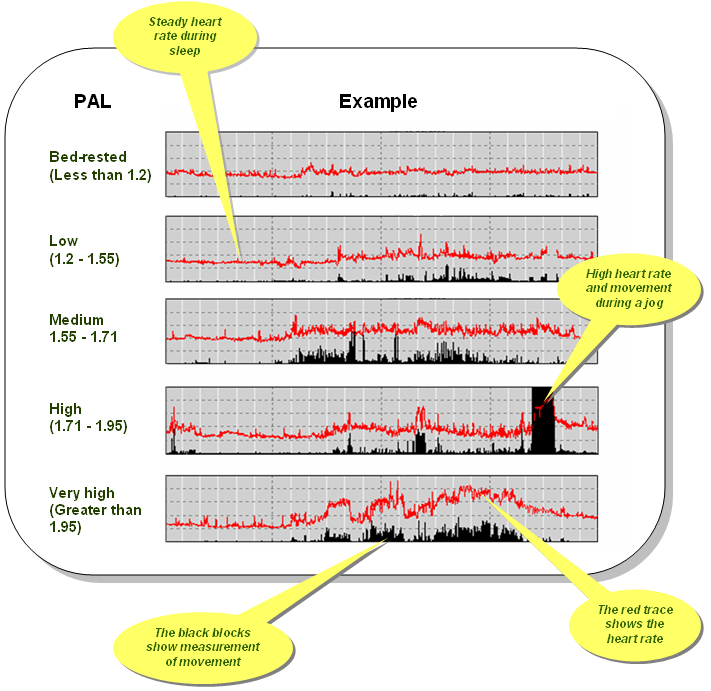
**

**How can I increase my physical activity level (PAL score)?**

Examples of what you can do to raise your physical activity level are shown in the table below. This tells you how much time you need to spend doing any one of these types of activities in a day to increase your daily PAL score by either 0.1 or 0.2 points:

| ***Activity*** | ***0.1 PAL points*** | ***0.2 PAL points*** |
| --- | --- | --- |
| ***Moderate housework*** | ***35 minutes*** | ***1 ¼ hours*** |
| ***Brisk walking*** | ***30 minutes*** | ***1 hour*** |
| ***Leisurely cycling*** | ***20 minutes*** | ***40 minutes*** |
| ***Light jogging*** | ***15 minutes*** | ***30 minutes*** |

**Jenny’s experience**

*[change to John for male participants]*

When Jenny received her feedback, the results showed that she had a physical activity level (PAL) of 1.4. She was surprised to find that this indicates a low level of activity. Being a busy parent who was often exhausted by the end of the day, she considered herself to be fairly active, and was disappointed about her result.

**Understanding the result**

When she thought more carefully about the main things that kept her busy, however, she realised that they didn’t involve much body movement or change in her heart rate or breathing. She noted down her daily activities for a week, and found that her typical day would be spent working at her desk in the office, driving the kids about, catching up on paperwork at home, making important phone calls, and organising her schedule. Although she was tired, she realised that it was often from having so much to think about, rather than from any physical activity.

**Setting goals**

Jenny decided that she would like to increase her level of physical activity in stages. Her first goal was to move from a low to a medium level, which meant increasing her PAL from 1.4 to at least 1.55. After some thought, she decided to set her target PAL at 1.6, which she felt was a manageable level.

**Making changes**

From the table, she chose an activity that she felt she could build into her daily routine, which in her case was walking. To reach her target of 1.6, she needed to increase her score by 0.2 points. According to the reference table, this was equivalent to an hour of brisk walking a day. As Jenny’s office was roughly a half-hour walk from her home, she decided to start walking to work and back instead of driving. She built this up gradually, and kept a record in her calender of what she was doing and how she was getting on. During the first week, she only walked on Tuesday and Thursday. By the fourth week, she was walking to work four or five days a week, and feeling much better.
